# Dr. Colwell D. Hale

**Published:** 1913-03

**Authors:** 


Dr. Colwell D. Hale, Cleveland University, 1877, one of the founders of the Syracuse Homoeopathic Hospital, died in Syracuse January 21, of cerebral haemorrhage, aged 71.

